# Evaluating the Lower Urinary Tract Syndrome with a Telemedicine Application: An Exploration of the Electronic Audiovisual Prostate Symptom Score

**DOI:** 10.3389/fsurg.2022.848923

**Published:** 2022-04-28

**Authors:** Ziyu Liu, Zhiyuan Guan, Hongyu Lan, Yan Zhao, Zhiming Ye, Daojun Lv, Qingfeng Yu, Ming Wang, Kaoqing Peng, Nanfei Fu, Giorgio Mazzon, Di Gu

**Affiliations:** ^1^ Department of Urology and Guangdong Key Laboratory of Urology, The First Affiliated Hospital of Guangzhou Medical University, Guangzhou, China; ^2^ Nanshan College, Guangzhou Medical University, Guangzhou, China; ^3^ Urology Department, San Bassano Hospital, Bassano del Grappa, Italy; ^4^ The First Clinical College, Guangzhou Medical University, Guangzhou, China

**Keywords:** benign prostatic hyperplasia (BPH), lower urinary tract symptoms (LUTS), telemedicine application, electronic symptom assessment, audiovisual symptom scale

## Abstract

**Background:**

The Visual Prostate Symptom Score (VPSS) is used for the assessment of lower urinary tract symptoms (LUTS). It is usually administered by general practitioners (GPs), but in these cases, outcomes do not seem to be reflecting the real conditions of a patient well, with consequent risks of misestimations and misinterpretations. We developed an electronic audiovisual version of VPSS (EPSS), a new symptom scale based on a telemedicine mobile light-based app. The aim of this study is to test and evaluate its reliability.

**Methods:**

We enrolled male patients aged between 50 and 80 years across 24 community-based healthcare facilities in Guangzhou, China. Patients were asked to complete the Chinese version of VPSS and EPSS before consultation with the urology specialists. Patients were divided into two groups based on age. First, we analyzed the rate of full understanding of EPSS using a chi-square test. Then, we analyzed the difference between each score of EPSS, VPSS, and outcomes measured by specialists, used as the reference score (RS). Finally, the outcomes were analyzed with the Spearman test and Bartlett test separately.

**Results:**

Seventy-nine male patients were included (mean age 70.42 years). Patients were divided into two groups: group 1 (>70 years, *n* = 40) and group 2 (<70 years, *n* = 39). The full-understanding rates in groups 1 and 2 were 50% and 64.1%, respectively. No significant differences were noted between groups (*p* = 0.206). A *t*-test was presented between each question of VPSS, EPSS, and RS. All questions did not display significant differences (*p* > 0.05); total scores from the three scales had no significant differences in the evaluation of LUTS. We further explored the variations of choices made by patients in different scales. Spearman’s test among VPSS, EPSS, and RS showed positive correlations, and coefficients of the total score were 0.92, 0.91, and 0.93 (*p* < 0.05).

**Conclusion:**

EPSS can be easily used in a significant number of patients and showed correlation with the VPSS and RS. Moreover, certain items resulted in better performance than VPSS. The results showed that EPSS could be a valuable option for both patients and GPs monitoring LUTS and particularly helpful when teleconsultations are considered, especially during the COVID-19 pandemic.

## Introduction

Lower urinary tract symptoms (LUTS) associated with benign prostatic hyperplasia (BPH) represent a prominent concern in aging men (1). In the last two decades, the assessment of male LUTS has progressively relied more on symptom scoring tools (2). In this context, the utilization of validated symptom score questionnaires has been recommended and widely accepted (3). The International Prostate Symptom Score (IPSS) was first developed and validated by the American Urological Association (AUA) in 1992, and its use has been recommended by EAU and AUA guidelines (4, 5).

Nevertheless, their frequent utilization, particularly among elderly people, is difficult. In fact, statements are presented with small font letters and complex sentences, with risks of misunderstandings and discrepancies between scores and patients’ real symptoms (6). Therefore, van der Walt developed a new Visual Prostate Symptom Score (VPSS) in 2011, with results at least as reliable as the IPSS in assessing subjective symptoms and the quality of life (QoL) (6).

In Chinese basic healthcare facilities, general practitioners (GPs) regularly use VPSS as an initial screening tool for the assessment of male LUTS. If necessary, patients are referred to urology specialists for further management. In this context, relying on efficient tools for screening becomes fundamental to guarantee an adequate level of care. Although not evident from published data, we found that VPSS filled with GPs and urologists’ assistance led to different results. This non-negligible deviation may lead to misestimating LUTS severity. We also noted that, in most circumstances, patients are unable to fulfill the questionnaire autonomously and healthcare workers have to provide support. Consequently, a bias may be involved as patients might be influenced by doctors’ words. We can speculate that the different explanation of the VPSS questions given by each doctor, especially when describing the urinary stream, is a possible font of discordant results. This issue can have effects on patient care and can have a detrimental effect on data collection for research purposes.

Telemedicine has been adopted in the pediatric field since 2011 (7) and in adults (8). Remote electronic consultations are convenient in many circumstances: they can provide timely access to specialist expertise, particularly in cases of patients living in remote areas or distant from with highly specialized centers (9). The advantage of electronic consultations becomes particularly obvious during the COVID-19 pandemic. This may reduce the number of patients’ hospital accesses and out-patient visits. Additionally, patients with chronic conditions unable to report to healthcare facilities can still receive an adequate and prompt assessment (10).

We made an audiovisual animated version of VPSS, creating a new electronic form of symptom scales based on a mobile light app (the exhibition picture and video are located in the Supplementary Material). Several innovations and breakthroughs in this electronic VPSS (EPSS) have been made: the voice broadcast can support patients (particularly those with visual impairment) in the correct completion; animations can provide a better understanding of specific questions, particularly when evaluating the urinary stream. Moreover, the LUTS assessment can be done without the presence of a doctor, reducing the number of patients in healthcare facilities. Additionally, GPs and specialists can monitor patients’ LUTS and evaluate remotely whether further management is required.

## Methods

### Patients’ Recruitment

In this study, male patients with LUTS were recruited from healthcare facilities in 24 communities in the city of Guangzhou, China. The study period was October 2021–November 2021. The inclusion criteria were (1) age between 50 and 80 years; (2) diagnosis of BPH, prostate cancer (watchful waiting and verified by biopsy); and (3) willingness to participate in this study. The exclusion criteria were (1) patients with urinary tract infections, (2) patients with prostatitis, and (3) withdrawal of consent to participate.

The study was approved by the ethical committee of the First Affiliated Hospital of Guangzhou Medical University and was performed in agreement with the tenets of the Declaration of Helsinki. All patients provided informed consent after receiving an explanation of the study.

Patients were divided into two groups based on age: group 1 (≥70 years) and group 2 (<70 years) to explore whether the EPSS is adaptable for patients of different age levels.

### EPSS and Scale Assessment

The EPSS is a new audiovisual animated version of the prostate symptom score based on a light app on a smartphone (mini-program). VPSS was built on WeChat and could be accessed through the QR code. The first three questions are presented with animations. We decided not to support QoL questions with animations as the figures are already exhaustive. Audio is available in three different languages during animations: Mandarin, Cantonese, and English. The animations were designed with our visual art consultant and supervised by urology specialists. The audios were created according to IPSS statements and confirmed by specialists for accuracy.

#### Scale Assessment

Two questionnaires were used in this research. The first one was the Chinese version of VPSS (printed by Astellas Pharma Inc.) In this version, the urinary stream was ranked as “normal, weak stream, slim stream, interruption, and dripping” in Chinese characters. These translated annotations were demonstrated to be a valid, reliable, and sensitive measure to assess Chinese females and males with LUTS (11, 12). The second questionnaire is the EPSS. In EPSS, we added animations to the first three questions (the QoL question was the same as the pictures in VPSS). Audio broadcasts were designed to illustrate the questions in Mandarin, Cantonese, and English. After filling the EPSS, we asked patients to rank their level of understanding as follows: totally understand (complete without assistance), partly understand (need of assistance to complete), and not understandable (not able to complete).

### Protocol

**Figure 1:**
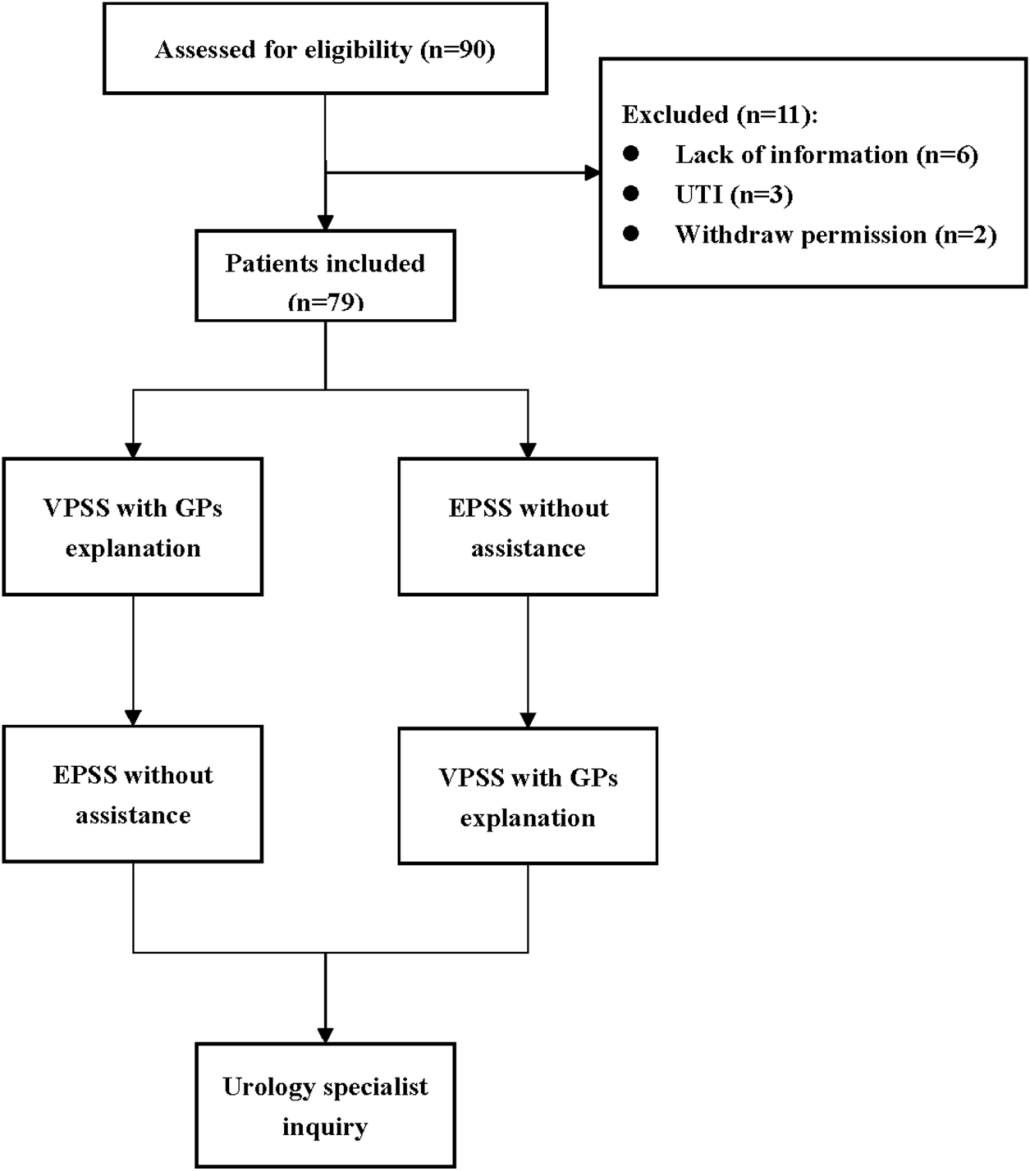
Protocol. A total of 90 patients were recruited, and 79 patients were finally included. We randomize the order of the two versions of the scale for each patient to minimize the potential bias.

Cases have been enrolled in out-patient clinics. Patients were asked to fill out two questionnaires based on their ongoing symptoms. The topic of the questions was not explained to patients beforehand. The VPSS was circulated and filled with the assistance of GPs. VPSS sheets were distributed and filled by each patient as per the standard protocol in basic health facilities. The EPSS was completed without any support. Patients used the mobile light app with their own smartphone and completed the questionnaire independently. Patients unable to utilize the smartphone have been supported by nursing staff with limited help (for instance, assistance during mobile phone utilization but no explanations have been provided to answer questions). In these cases, an understanding level of “totally understand” could not be assigned. Subsequently, patients have been reviewed by trained urology specialists. A new VPSS was filled by the specialist during a well-rounded consultation with patients (recorded as reference score, RS). To minimize the potential bias caused unconsciously, the urology specialist was asked to provide an explanation according to the descriptions in IPSS and intentionally minimize the extra instruction or leading patients’ opinion. Results of the three questionnaires were compared. The study process is summarized in Figure 1. Each patient completed the whole process on the same day. The order of the two questionnaires was randomized to minimize the risk of potential biases.

#### Statistical Analyses

Statistical analyses were performed with R4.1.2. Patients were divided into two groups based on age. Between two groups, the chi-square test was used to analyze the rate of the full-understanding level of EPSS. We analyzed the difference between each score of EPSS, VPSS, and RS. Correlations and differences between EPSS, VPSS, and RS performed by specialists were analyzed with the Spearman test and Bartlett test. *p* < 0.05 was considered significant.

## Results

A total of 79 patients were finally included in the study; baseline characteristics are shown in Table 1. The mean age of patients was 70.42 years (SD ± 6.67, range 52–86). The mean VPSS score was 12.57 (SD ± 3.66), and the mean EPSS score was 12.95 (SD ± 3.77). The mean RS was 12.43 (SD ± 3.79). Seventy-four patients had a PSA test, and the mean PSA value was 9.63 ng/mL (SD ± 7.75) (Table 2).

**Table 1:** Baseline characteristics of patients enrolled.

	Group 1	Group 2	*p-*Value
*n*	39	40	
Age [mean (SD)]	65.440 (4.91)	75.28 (4.06)	<0.001
Height [mean (SD)]	166.74 (5.86)	165.34 (6.52)	0.317
Weight [mean (SD)]	64.870 (9.98)	61.62 (10.90)	0.171
BMI [mean (SD)]	23.390 (3.77)	22.44 (3.15)	0.229
Exercises per week (%)			0.780
0–1	4 (10.3)	3 (7.5)	
2–3	13 (33.3)	11 (27.5)	
4–5	7 (17.9)	6 (15.0)	
6–7	15 (38.5)	20 (50.0)	
Smoking (%)			0.420
Current	14 (35.9)	9 (22.5)	
Never	19 (48.7)	24 (60.0)	
Used to	6 (15.4)	7 (17.5)	

**Table 2:** VPSS score, EPSS score, RS, and comprehension rate between two groups.

	VPSS					EPSS						RS				
	Q1	Q2	Q3	Q4	Total	Q1	Q2	Q3	Q4	Total	Comprehension rate (%)	Q1	Q2	Q3	Q4	Total
Age >70 (*n* = 40)	5.42	2.75	2.75	1.8	12.72	5.3	2.98	2.88	2.02	13.18	51.28	5.55	2.85	2.38	1.63	12.54
Age ≤70 (*n* = 39)	5.46	2.2	2.85	1.9	12.41	5.46	2.2	2.9	2.15	12.72	65.79	5.51	2.13	2.67	2.1	12.41
All (*n* = 79)	5.44	2.48	2.8	1.85	12.57	5.38	2.59	2.89	2.09	12.95	56.96	5.53	2.49	2.52	1.89	12.43

According to age, 40 patients were enrolled in group 1 (>70 years) and 39 in group 2 (<70 years). In group 1, 20 patients (50.0%) could fully understand EPSS and complete the study without help. The fully understanding rate in group 2 was 64.1% (25/39). The chi-square test did not show a significant difference in the full-understanding rate (*p* = 0.206).

A *t*-test was used for each question of VPSS, EPSS, and RS. All questions did not display significant differences (*p* > 0.05). These results showed that the total score of the three scales had no significant differences in evaluating LUTS.

**Figure 2:**
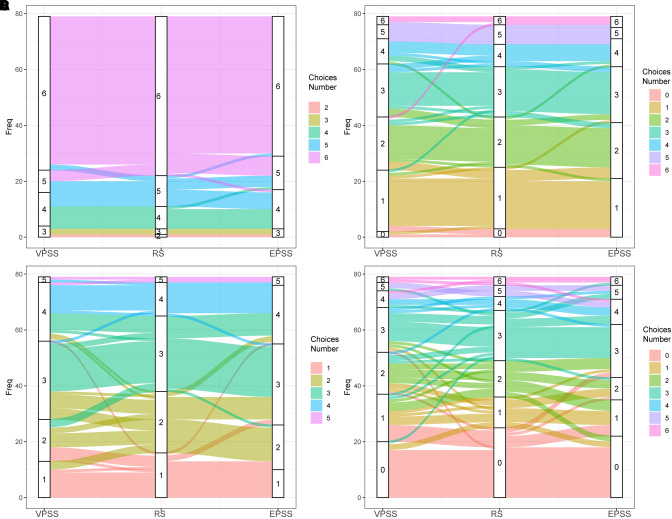
Sankey diagram showing the different choices patients made among VPSS, RS, and EPSS in (**A**) question 1, (**B**) question 2, (**C**) question 3, and (**D**) question 4. By comparing the flows on the two sides, we could see which scale performed better. No obvious differences were seen: (**A**) both VPSS and EPSS sides were very neat and (**D**) both sides were chaotic. In (**B**,**C**), EPSS performed better with less and thinner chaos flows.

**Figure 3:**
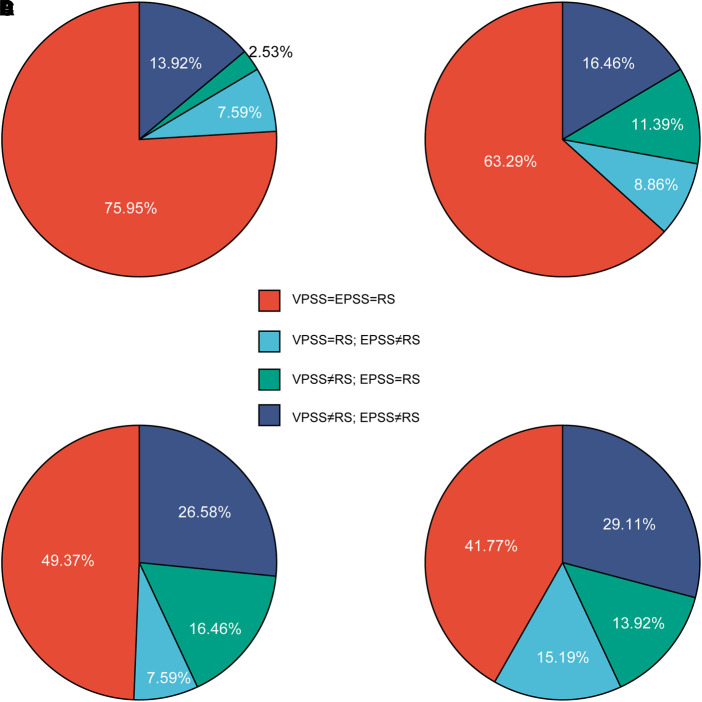
Pie charts to analyze specific accuracy. (**A**)–**D**) represented questions 1 to 4. We compared the patients’ answers in VPSS and EPSS with the reference score (RS) to see which scale captured more answers identical to RS while the other did not. In (**A**), both scales collected most of the answers identical to RS (75.95%), showing no prominent advantages. In (**A**) and (**B**), more than half of the answers collected on both scales were the same (75.95%% and 63.29%, respectively). However, panels (**C**) and (**D**) showed a sharp decrease (red part). Especially in (**C**), more identical answers were captured by EPSS independently (26.58%), indicating that EPSS shows advantages to VPSS in question 3. It proves the positive effect of animation. In (**D**), VPSS and EPSS showed approximate and poor specific accuracy (15.19% and 19.92%, respectively), confirming the result of the Sankey diagram (Figure 4).

To explore whether patients would change their minds when filling different questionnaires, we did further analysis. We found that variations in responses existed in three different scales in the Sankey diagram (Figure 2), showing that EPSS performed better than VPSS in questions 2 and 3. The specific accuracy of VPSS and EPSS was further analyzed and presented in pie charts (Figure 3). These results reflected the higher sensitivity of EPSS in distinguishing symptoms, which did not distinguish well through inquiry, and highlighted the advantages of EPSS to VPSS.

**Figure 4:**
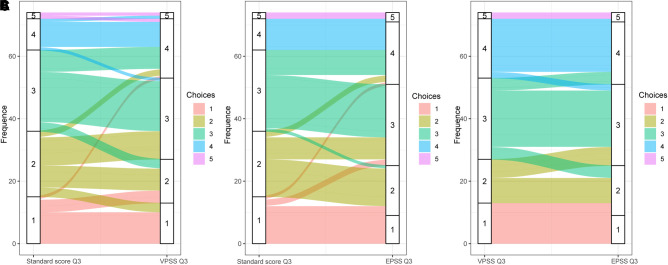
The Sankey diagram showing the different choice patients made of question 3 in (**A**) RS and VPSS, (**B**) RS and EPSS from doctors, and (**C**) VPSS and EPSS. Choices No. 1 to No. 5 represent normal, weak stream, slim stream, interruption urination, and dripping. The flow of colors reflects the different choices patients made in VPSS, EPSS, and RS, representing the patients changed their mind with different scales. The width of flow represents the amount. Both VPSS and EPSS revealed more “urinary interruption” situations (the No. 4 choice) than doctors. The flow in (**A**) is obviously more chaotic and wider than the flow in (**B**), indicating a prominent advantage of EPSS in question 3. In (**C**), flows of VPSS ended in separated EPSS choices. It shows that EPSS differentiates the “normal” (No. 1 choice) and “weak stream” (No. 2 choice) conditions better than VPSS.

**Figure 5:**
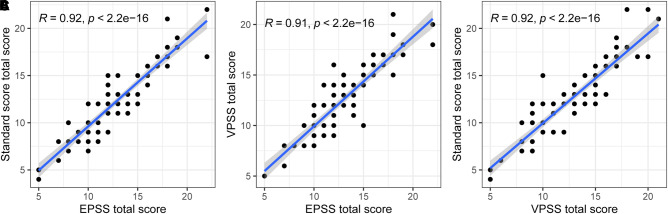
Correlation between (**A**) EPSS total score and RS, (**B**) EPSS total score and VPSS total score, and (**C**) VPSS total score and RS.

Spearman’s test was performed among total scores of VPSS, EPSS, and RS. A positive correlation was present between each question, and *p*-values are presented in Table 3 and Figures 4, 5. Coefficients of the total scores between EPSS and RS, EPSS and VPSS, and VPSS and RS were 0.92, 0.91, and 0.93, respectively (Table 4). In conclusion, the result showed that EPSS and VPSS had the same accuracy in estimating LUTS compared with RS and VPSS has been proved to be a reliable tool to evaluate LUTS in clinical diagnosis and treatment.

**Table 3:** Spearman’s correlation of each question between VPSS, EPSS, and RS.

	Spearman correlation	*p-*Value
EPSS and RS Q1	0.83	<0.05
EPSS and RS Q1	0.92	<0.05
VPSS and RS Q1	0.88	<0.05
EPSS and RS Q2	0.91	<0.05
EPSS and VPSS Q2	0.89	<0.05
VPSS and RS Q2	0.88	<0.05
EPSS and RS Q4	0.85	<0.05
EPSS and VPSS Q4	0.82	<0.05
VPSS and RS Q4	0.81	<0.05

**Table 4:** Spearman’s correlation of total score between VPSS, EPSS, and RS.

	Spearman correlation	*p*-Value
Total EPSS and RS	0.92	<0.05
Total EPSS and VPSS	0.91	<0.05
Total VPSS and RS	0.93	<0.05

## Discussion

LUTS secondary to BPH is a common condition in aging men. The IPSS was first developed and validated by the AUA in 1992 (13). It was designed as a self-administered questionnaire for patients, and it represented a trend toward the conversion of subjective symptoms into a quantifiable objective index, able to weigh the severity of clinical conditions (4).

Nevertheless, a certain number of patients may have significant difficulties in completing the questionnaire, particularly those individuals with a lower education level. The result is poorer reliability of this tool (6, 14). Moreover, elderly people may struggle in understanding the questionnaire, especially if suffering from visual impairment (1). Therefore, van der Walt developed a new VPSS in 2011 (6), a four-item questionnaire evaluating LUTS through pictures and brief descriptions, covering urinary frequency, stream, and quality of life (QoL).

In modern management of LUTS, the symptom scales are used for patients’ assessment/screening, choice of treatments, and reevaluation during follow-ups (2). Variations of IPSS may reflect patients’ response to treatment and expectations (5). So, the symptom scales should be as reliable as possible since they may affect the whole management plan. For example, during follow-ups, a deteriorated symptom score will need doctors to reassess or consider alternative therapy. In the field of research, a reliable and valid outcome measurement is critical for data comparison. Additionally, besides patients’ care, they represent important parameters for insurers and regulators. As the only subjective representation of patient’s symptoms and comfort, the scales should provide reproducible results and minimize differences dependent on different clinicians (13). These are well-known drawbacks of current questionnaires.

Telemedicine has been used for remote appointments, video consultations, patients’ education, and so on (8–10). Self-assessments represent a new extension of telemedicine. The utilization of electronic scales was proven to optimize medical out-patient care. For example, patients can start their assessment while waiting in the waiting room. As a result, more time can be dedicated to each patient (15). In the literature, a visual self-assessed questionnaire for skin disease also showed good reliability (16). Similarly, cartoon questionnaires were considered a more effective and standardized tool for children (17). EPSS is in line with these principles and, we believe, can be a very helpful tool in different scenarios.

In our out-patient setting, patients with LUTS are routinely assessed after an initial screening with GPs. We observed that there were non-negligible deviations in VPSS scores. During these appointments, GPs frequently support patients in the completion of the questionnaire.

These phenomena highlighted a few concerns. First, some patients were not confident in completing the questionnaire independently of the VPSS (especially the elderly). Many patients might also complain to require a long time to understand and answer appropriately, as pictures may not be clear. For this reason, they might be tempted to provide a response that is not reflecting real symptoms. Second, we noticed that the elderly people tend to prefer a verbal explanation instead of reading themself.

For these reasons, integrating VPSS with vocal broadcasts may provide advantages, avoiding the misunderstanding or misjudging caused by different doctors. Most importantly, the broadcast only provides basic and necessary descriptions, without any other extra instructions or hints. This characteristic erased the bias mentioned.

VPSS administered by the GPs and EPSS show a good correlation with RS in questions 1 and 2, indicating that better understanding could be achieved if an explanation is provided (with or without doctors’ help). These data suggest that patients can easily fulfill audible questionnaires compared to traditional written versions. Adding animations to questions 1 and 2 lies mainly in the enrichment of the presentation—a more vivid and varied format can increase patient interest and compliance. As a result, Spearman’s correlation coefficients between the three scales approach 1. This indicated that EPSS had a close efficiency in estimating LUTS with VPSS.

A clear and reproducible description of the urinary stream was one of our main expectations and a significant breakthrough. Urination is determined by consecutive actions instead of a static state, so we presumed that animations, showing the whole urination process, would make a better presentation than a graph. For example, a dotted line was used in VPSS for interrupted urinary flow, but this kind of stream does not exist in the real world. This dilemma brought us to develop an animated version. On the other hand, in the current version of EPSS, viewing the urinary stream animations takes seconds and some patients may be tempted by choosing an option before waiting the end of all videos. In future EPSS developments, showing different types of urinary streams at the same time may be the optimal solution.

According to our results, question 3 (urinary stream) shows some interesting phenomena, as expected. The urinary streams are described in VPSS as “normal, weak stream, slim stream, interruption, and dripping.” However, in patient’s mind, a “weak stream” usually is associated with a “slim stream,” thus making it hard for patients to decide. We noticed that when patients hesitated to choose stream 2 or 3, a doctor was often involved in the decision-making process, leading to several biases (13).

Using EPSS may therefore reduce the influence of doctors on patients’ choices because it only contains specialist-liked questions that are critical to the assessment but clear from additional doctor’s explanations or interpretations that may interfere with the patient’s feelings (just like how we required the specialist involved in the study). As our research showed (Figure 3), both VPSS and EPSS revealed more “urinary interruptions” (the No. 4 choice) than doctors. Then, EPSS captured more identical answers to RS than VPSS (Figure 3). Even more, EPSS differentiates the “normal” (No. 1 choice) and “weak stream” (No. 2 choice) better than the RS assigned by doctors and even better than VPSS (Figure 4). This indicated that the sensitivity of EPSS in question 3 was even higher than the urology specialists’ inquiry. These results, we believe, are determined by the introduction of animations. In this way, EPSS could also assist urology specialists’ evaluations.

This study could not ascertain which questionnaire was more reliable and a more objective reflection of the true extent of the patient’s LUTSs. Therefore, further studies should be conducted to explore this unknown by adding urinary volume as an objective standard. Also, there is room for improvement in the animations, such as highlighting the differences in the streamlines and adding shoes to indicate “dripping.” As a supplement, it will be worth exploring whether “urinary retention and in need for catheterization” should also be included in the EPSS.

In China, when needing assessment for acute and chronic medical conditions, patients are advised to report to primary healthcare services first. GPs represent the first screening level, playing an important role in patients’ management. Thus, another function of a practical scale is to offer a valid first-line screening, identifying patients to address for specialists’ evaluation. EPSS can also offer patients an adequate self-assessment, reducing access to primary healthcare services and hospitals, favoring also remote counseling. This fact is becoming particularly important, especially as a consequence of the COVID-19 pandemic across the world. The EPSS could also provide benefits in both metropolitan and rural areas, minimizing the problem of long travel distances and additional costs for patients.

Telemedicine surely has some potential drawbacks, like the disparity of access to technology could happen in rural and inner cities (9). However, in our study, the significance of exploring EPSS lies precisely in helping people who face difficulties in accessing specialist assessments through GPs. Additionally, Spearman’s correlation coefficients between scales are approaching 1. This may indicate that EPSS and VPSS have a comparable efficiency in estimating LUTS.

Another significant and unavoidable problem is that EPSS cannot be used by patients uncomfortable with modern technologies (smartphone, internet platforms, and so on), a common phenomenon among elderly people (18). To minimize this factor, we asked for support to our art and design consultants and paid extra attention to details, including the size of the animations, their comprehensibility, and so on.

This is the first article in the literature introducing and investigating the EPSS questionnaire. However, there are several limitations to our study. First, the sample size was limited. Second, some relevant clinical data were not present, including ongoing medical treatments, comorbidities, urine flow rates, and postvoiding residuals. Third, we did not divide patients into first-visit and follow-up groups. Follow-up patients may be familiar with symptom questionnaires, resulting in a better understanding level. Moreover, patients who fulfilled all questionnaires on the same day may potentially influence the results. Finally, VPSS has not been filled without GP support; therefore, we cannot evaluate the level of understanding of patients if not supported by medical staff.

Nevertheless, the model is an innovation and the results are promising. Further studies should be conducted in larger cohorts of patients, including also non-Chinese populations.

## Conclusion

We developed and tested EPSS, an innovative electronic audiovisual version of VPSS. It demonstrated to be well comprehended and accepted by patients with LUTS, and it also showed a significant correlation with the VPSS and assessment by urology specialists. Moreover, certain aspects of LUTSs resulted in being better investigated by EPSS if compared to VPSS. EPSS represents a valid option for the assessment of male LUTS and may be particularly indicated for telemedicine services.

## Data Availability

The raw data supporting the conclusions of this article will be made available by the authors without undue reservation.

## References

[1] Cam K. BPH: How useful is a visual prostate symptom score for patients? Nat Rev Urol. (2011) 8(10):536–7. 10.1038/nrurol.2011.137 21931343

[2] Gratzke C Bachmann A EAU guidelines on the assessment of non-neurogenic male lower urinary tract symptoms including benign prostatic obstruction. Eur Urol. (2015) 67(6):1099–109. 10.1016/j.eururo.2014.12.038 25613154

[3] Bonnal JL Irani J Management of bladder cancer associated with prostate cancer. Prog Urol. (2003) 13(6):1286–9. 15000300

[4] Mottet N van den Bergh R EAU-EANM-ESTRO-ESUR-SIOG guidelines on prostate cancer—2020 update. Part 1: screening, diagnosis, and local treatment with curative intent. Eur Urol. (2021) 79(2):243–62. 10.1016/j.eururo.2020.09.042 33172724

[5] Lerner LB McVary KT Management of lower urinary tract symptoms attributed to benign prostatic hyperplasia: AUA guideline part I—initial work-up and medical management. J Urol. (2021) 206(4):806–17. 10.1097/JU.0000000000002183 34384237

[6] van der Walt CL Heyns CF Prospective comparison of a new visual prostate symptom score versus the international prostate symptom score in men with lower urinary tract symptoms. Urology. (2011) 78(1):17–20. 10.1016/j.urology.2011.01.065 21550646

[7] Canon S Shera A A pilot study of telemedicine for post-operative urological care in children. J Telemed Telecare. (2014) 20(8):427–30. 10.1177/1357633X14555610 25316038

[8] Park ES Boedeker BH The initiation of a preoperative and postoperative telemedicine urology clinic. Stud Health Technol Inform. (2011) 163:425–7. 10.3233/978-1-60750-706-2-425 21335833

[9] Miller A Rhee E The current state of telemedicine in urology. Med Clin North Am. (2018) 102(2):387–98. 10.1016/j.mcna.2017.10.014 29406066

[10] Hong Z Li N Telemedicine during the COVID-19 pandemic: experiences from Western China. J Med Internet Res. (2020) 22(5):e19577. 10.2196/19577 32349962 PMC7212818

[11] Choi EP Lam CL Validation of the International Prostate Symptom Score in Chinese males and females with lower urinary tract symptoms. Health Qual Life Outcomes. (2014) 12:1. 10.1186/1477-7525-12-1 24382363 PMC3883473

[12] Yao MW Green J. How international is the International Prostate Symptom Score? A literature review of validated translations of the IPSS, the most widely used self-administered patient questionnaire for male lower urinary tract symptoms. Low Urin Tract Symptoms. (2022) 14(2):92–101. 10.1111/luts.12415 34734477

[13] Barry MJ Fowler FJ The American Urological Association Symptom Index for benign prostatic hyperplasia. J Urol. (2017) 197(2S):S189–97. 10.1016/j.juro.2016.10.071 28012747

[14] Rodrigues NNJ de Lima ML Latin American study on patient acceptance of the International Prostate Symptom Score (IPSS) in the evaluation of symptomatic benign prostatic hyperplasia. Urology. (1997) 49(1):46–9. 10.1016/S0090-4295(96)00372-X 9000184

[15] Lam SCV Kastner M Development process and patient usability preferences for a touch screen tablet-based questionnaire. Health Informatics J. (2020) 26(1):233–47. 10.1177/1460458218824749 30672358

[16] Cazzaniga S Naldi L Validation of a visual-aided questionnaire for the self-assessment of hidradenitits suppurativa. J Eur Acad Dermatol Venereol. (2018) 32(11):1993–8. 10.1111/jdv.15050 29729101

[17] Qiu S Xia Y Using a cartoon questionnaire to improve consent process in children: a randomized controlled survey. Pediatr Res. (2021) 90(2):411–8. 10.1038/s41390-020-01227-2 33203966

[18] Ang S Lim E Health-related difficulty in internet use among older adults: correlates and mediation of its association with quality of life through social support networks. Gerontologist. (2021) 61(5):693–702. 10.1093/geront/gnaa096 32744310

